# Nicosulfuron Degradation by an Ascomycete Fungus Isolated From Submerged *Alnus* Leaf Litter

**DOI:** 10.3389/fmicb.2018.03167

**Published:** 2018-12-19

**Authors:** Louis Carles, Florent Rossi, Pascale Besse-Hoggan, Christelle Blavignac, Martin Leremboure, Joan Artigas, Isabelle Batisson

**Affiliations:** ^1^Laboratoire Microorganismes: Génome et Environnement, CNRS, Université Clermont Auvergne, Clermont-Ferrand, France; ^2^Institut de Chimie de Clermont-Ferrand, CNRS, Sigma Clermont, Université Clermont Auvergne, Clermont-Ferrand, France; ^3^Centre Imagerie Cellulaire Santé, Université Clermont Auvergne (UCA PARTNER), Clermont-Ferrand, France

**Keywords:** ascomycete fungus, herbicide, sulfonylurea, degradation, co-metabolism, natural substrata, *Plectosphaerella cucumerina*

## Abstract

Nicosulfuron is a selective herbicide belonging to the sulfonylurea family, commonly applied on maize crops. Its worldwide use results in widespread presence as a contaminant in surface streams and ground-waters. In this study, we isolated, for the first time, the *Plectosphaerella cucumerina* AR1 nicosulfuron-degrading fungal strain, a new record from *Alnus* leaf litter submerged in freshwater. The degradation of nicosulfuron by *P. cucumerina* AR1 was achieved by a co-metabolism process and followed a first-order model dissipation. Biodegradation kinetics analysis indicated that, in planktonic lifestyle, nicosulfuron degradation by this strain was glucose concentration dependent, with a maximum specific degradation rate of 1 g/L in glucose. When grown on natural substrata (leaf or wood) as the sole carbon sources, the *Plectosphaerella cucumerina* AR1 developed as a well-established biofilm in 10 days. After addition of nicosulfuron in the medium, the biofilms became thicker, with rising mycelium, after 10 days for leaves and 21 days for wood. Similar biofilm development was observed in the absence of herbicide. These fungal biofilms still conserve the nicosulfuron degradation capacity, using the same pathway as that observed with planktonic lifestyle as evidenced by LC-MS analyses. This pathway involved first the hydrolysis of the nicosulfuron sulfonylurea bridge, leading to the production of two major metabolites: 2-amino-4,6-dimethoxypyrimidine (ADMP) and 2-(aminosulfonyl)-*N*,*N*-dimethyl-3-pyridinecarboxamide (ASDM). One minor metabolite, identified as 2-(1-(4,6-dimethoxy-pyrimidin-2-yl)-ureido)-*N,N*-dimethyl-nicotinamide (N3), derived from the cleavage of the C-S bond of the sulfonylurea bridge and contraction by elimination of sulfur dioxide. A last metabolite (N4), detected in trace amount, was assigned to 2-(4,6-dimethoxy-pyrimidin-2-yl)-*N,N*-dimethyl-nicotinamide (N4), resulting from the hydrolysis of the N3 urea function. Although fungal growth was unaffected by nicosulfuron, its laccase activity was significantly impaired regardless of lifestyle. Leaf and wood surfaces being good substrata for biofilm development in rivers, *P. cucumerina* AR1 strain could thus have potential as an efficient candidate for the development of methods aiming to reduce contamination by nicosulfuron in aquatic environments.

## Introduction

Nicosulfuron (2-[(4,6-dimethoxypyrimidin-2-yl)carbamoylsulf-amoyl]-*N,N*-dimethylpyridine-3-carboxamide) is a sulfonylurea class herbicide used worldwide as a post-emergence herbicide to protect maize crops from weeds. It inhibits acetolactate synthase (ALS) enzyme activity, a key enzyme involved in the branched-chain amino acid biosynthesis (Schloss, 1990), which results in the inhibition of plant growth. Despite the low agronomic dose recommended for nicosulfuron in crops (in Europe, 60 g active ingredient/ha; CE 1107/2009), this molecule is frequently detected in surface and ground-waters due to its high mobility, its Groundwater Ubiquity Score (GUS) being of 3.34 (Pfeiffer, 2010). This transfer can be explained by the high solubility (>7 g/L at pH ≥ 6.5) and low K_d_ coefficient of the molecule (ranging from 0.14 to 2.15 L/kg, Gonzalez and Ukrainczyk, 1996, 1999; Oliveira et al., 2001; Regitano and Koskinen, 2008; Trigo et al., 2014; Azcarate et al., 2015). The nicosulfuron environmental concentrations found in various surface waters from Canada, United States, and Europe, averaging 0.3–0.5 μg/L, are non-negligible (Battaglin et al., 2000; de Lafontaine et al., 2014; Moschet et al., 2014), the highest amounts detected peaking up to 3.29 μg/L (Battaglin et al., 2009). Overall, the high detection frequency of nicosulfuron in surface waters implies chronic exposure of aquatic microbial communities and eventually a set of adaptations regarding its use by microbes.

Responses of aquatic fungi to organic contaminants are sequential regarding exposure time and can take from hours to weeks. After an acute exposure to the pesticide, the first fungal responses consist in the oxidative attack of the molecule both in the intracellular (i.e., cytochrome P450 monooxygenases) and/or extracellular (i.e., peroxidases and laccases) spaces, followed by a methylation or conjugation process which improve the molecule solubility as well as its release out of the cell (Krauss et al., 2011). After a chronic exposure, fungi can mineralize the molecule with more or less success.

Regarding the sensitivity of fungi to nicosulfuron, most studies have been performed in soils. For instance, soil fungi are sensitive to nicosulfuron when repeatedly applied at dose rates higher than the recommended one, probably because ALS genes are also present in numerous fungal species (Karpouzas et al., 2014). In contrast, increasing levels of nicosulfuron exposure have been shown to increase the bacterial abundance and diversity in soil (Petric et al., 2016). This tolerance to nicosulfuron seems to be widespread in soil bacteria, mainly among Firmicutes and Actinobacteria. In aquatic microbial communities, responses to nicosulfuron are rather different comparing to those observed in soils (Carles et al., 2017b). While chronic exposure to nicosulfuron enhances fungal diversity in aquatic microbial communities associated with leaf litter, the diversity of bacteria was severely impaired. At the same line, the pre-exposure history of these aquatic microbial communities to contamination played a significant role in their ability to biodegrade nicosulfuron (Carles et al., 2017b).

Degradation of nicosulfuron can require up to 70 days in natural aquatic environments (Cessna et al., 2015), both abiotic and biotic degradation processes co-occurring. Chemical hydrolysis of the sulfonylurea linkage has been described as the main abiotic degradation process (Sarmah and Sabadie, 2002), its rate being greater as pH of the medium decreases (Berger and Wolfe, 1996). This phenomenon was also observed for *Penicillium oxalicum* YC-WM1 where nicosulfuron degradation was due to medium acidification resulting from oxalate secretion by the fungal strain (Feng et al., 2017). Two degradation products are usually formed: 2-amino-4,6-dimethoxypyrimidine (ADMP) and 2-(aminosulfonyl)-*N,N*-dimethyl-3-pyridinecarboxamide (ASDM), the latter being able to cyclize at basic pH (Sarmah and Sabadie, 2002). Besides, five different photo-products have been identified during nicosulfuron photodegradation in aqueous media (Benzi et al., 2011), though the contribution of this process to total abiotic degradation of the molecule seems to be of minor importance (EFSA, 2007). Regarding biotic degradation, nine bacterial [*Oceanisphaera psychrotolerans* LAM-WHM-ZC (Zhou et al., 2017), *Bacillus subtilis* YB1 (Yang et al., 2008; Lu et al., 2012), *Ochrobactrum* sp. ZWS16 (Zhao et al., 2015b), *Rhodopseudomonas sp.* J5-2 (Zhang et al., 2011), *Alcaligenes faecalis* ZWS11 (Zhao et al., 2015a), *Klebsiella* sp. Y1 (Wang et al., 2016), *Serratia marcescens* N80 (Zhang et al., 2012), *Pseudomonas fluorescens* SG-1 (Carles et al., 2017a) and *Pseudomonas nitroreducens* strain NSA02 (Zhao et al., 2018)], and three fungal [*Talaromyces flavus* LZM1 (Song et al., 2013), *Aspergillus niger* YF1 (Yang et al., 2008; Lu et al., 2012) and *Penicillium oxalicum* YC-WM1 (Feng et al., 2017)] nicosulfuron-degrading strains have been described in the literature. In most cases, the metabolites identified were ADMP and ASDM, suggesting similar degradation pathways. All these strains have been isolated from environments subjected to high anthropogenic pressure (i.e., wastewater treatment plants, agricultural soils), whereas no data are available about degrading ability of strains exposed to nicosulfuron in final ecological receptors such as river ecosystems.

The present study investigates the capacity of a fungal strain of *Plectosphaerella cucumerina* AR1, isolated from submerged leaves in a forested river, to degrade nicosulfuron. The influence of lifestyle, accessible carbon source use and activity of the strain during the dissipation process of the herbicide was assessed.

## Materials and Methods

### Chemicals and Media

Nicosulfuron (Pestanal, purity 99.6%) and ADMP (2-amino-4,6-dimethoxypyrimidine, purity 98.0%) were purchased from Sigma Aldrich (France), and ASDM (2-(aminosulfonyl)-*N,N*-dimethyl-3-pyridinecarboxamide, purity 98%) from J and K Scientific (Germany).

Malt extract and Sabouraud chloramphenicol agar media were purchased from Sigma Aldrich (France). Potato dextrose agar (PDA) medium was obtained from Biomérieux (France). Mineral salt medium (MSM) was composed of (/L): 1 g (NH_4_)_2_HPO_4_, 1 g KH_2_PO_4_, 1 g KNO_3_, 0.2 g MgSO_4_.7H_2_O, 0.1 g NaCl, 20 mg CaCl_2_, 5 mg FeSO_4_.7H_2_O, 1 mL of a salt stock solution and 1 mL of a vitamin stock solution. The salt stock solution contained (/L) 20 g boric acid, 18 g MnSO_4_.H_2_O, 2 g ZnSO_4_, 1 g CuSO_4_, 2.5 g Na_2_MoO_4_, 10 mg Co(NO_3_)_2_. The vitamin stock solution contained (/L) 2 mg biotin, 5 mg thiamine-HCl. The glucose-mineral salt medium (GSM) was obtained by addition of glucose (10, 5 or 1 g/L) in MSM. All the media were supplemented by chloramphenicol (0.5 g/L).

### Isolation and Identification of a Nicosulfuron-Degrading Fungal Strain

**FIGURE 1:**
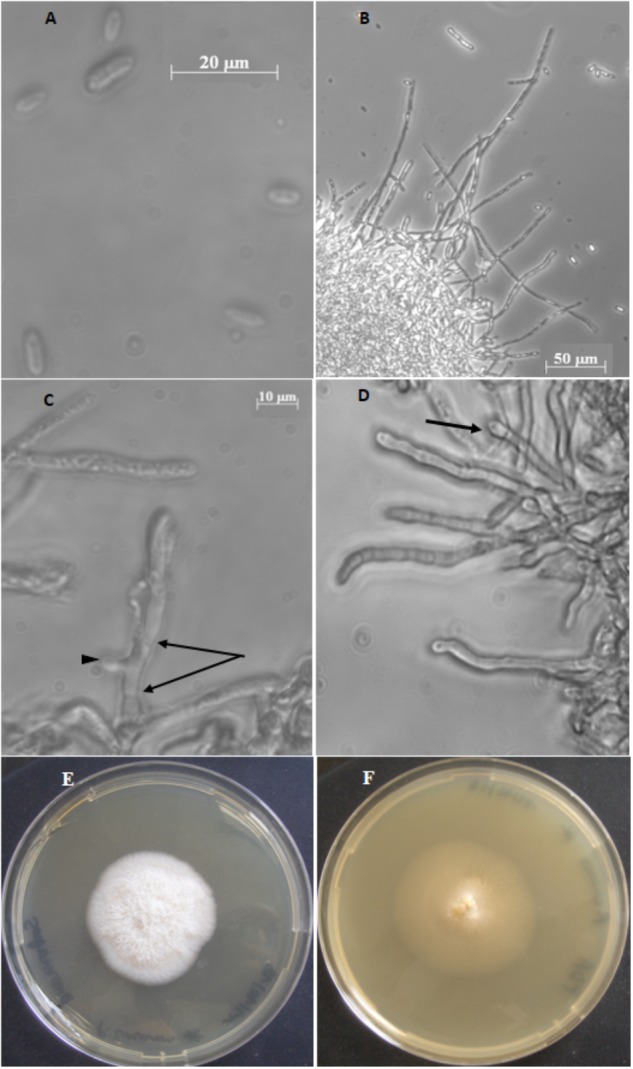
*Plectosphaerella cucumerina* AR1 strain. **(A)** Spores. **(B)** Mycelium with hyphae. **(C)** Septa hypha (arrows) and budding of hypha (arrow head). **(D)** Hyphal with phialide (arrow). **(E)** Colony growth on Sabouraud chloramphenicol agar medium. **(F)** Colony growth on PDA medium.

The isolation of fungal species was carried out on a nicosulfuron-degrading aquatic microbial community colonizing *Alnus glutinosa* (L.) Gaertn. leaf species (henceforth referred to *Alnus* in the text (Carles et al., 2017b)). The isolation was performed according to Artigas et al. (2017). Briefly, sporulation was induced in *Alnus* communities exhibiting nicosulfuron degradation. Among the spores obtained, a single spore morphotype (fusoid-type; Figure 1A) was physically isolated using glass micropipettes under a microscope (Leica DM IRB, Leica Microsystems, Wetzlar, Germany) and cultivated. Colony morphology was determined on culture grown on PDA or Sabouraud chloramphenicol agar media after 14 days of incubation at 23°C in the dark. Mycelium and spores were observed and photographed under an inverted microscope (Ziess, Axiovert 200M). A 10 μL spore suspension (containing ca. 15 spores) was then used for germination in 20 mL of malt extract 1% (pH 6.5) containing 0.5 mg/mL of chloramphenicol for 15 days at 28°C.

The identification of the fungal species was performed through DNA extraction from the fungal mycelium using the Fast DNA SPIN Kit for soil (MP Biomedicals, United States) and following the manufacturer’s instructions. Extracted DNA was then amplified by targeting from the fungal 18S to the 28S regions [using the primer pairs ITS 5 (5′-GGAAGTAAAAGTCGTAACAAGC-3′) (White et al., 1990) and NL 4 (5′-GGTCCGTGTTTCAAGAC-3′) (O’Donnell and Gray, 1995)]. The amplification reaction was carried out in a total volume of 50 μL containing 200 μM of each deoxyribonucleotide triphosphate (dNTP), 0.2 μM of each primer, 1 X PCR buffer containing 2.5 mM MgCl_2_, 0.3 U of Taq polymerase (Eurobio) and 50 ng of genomic DNA. Polymerase chain reactions (PCR) were performed as follows: 5 min at 95°C, followed by 35 steps [1 min. at 95°C, 2 min. at 52°C and 1 min. at 72°C] and a final elongation step at 72°C for 7 min. PCR amplicon was then sequenced (MWG – Biotech). The sequence obtained (1118 bp) was compared against NCBI sequences database using BLAST and deposited in GenBank under the Accession No: MK079567.

### Biodegradation of Nicosulfuron

#### In Planktonic Lifestyle Without or With Glucose as the Carbon Source

The 100 μM nicosulfuron biodegradation capacity of the strain was determined by inoculating from 0.25 to 0.35 mg of mycelium in 100 mL of mineral medium containing (GSM with 1, 5 or 10 g/L) or not (MSM) glucose in 250 mL flasks. The flasks were incubated at 28°C on an orbital shaker at 150 rpm in the dark to avoid photolysis. Non-inoculated media served as abiotic controls. Flasks inoculated only with the fungal strain were used as mycelium growth control.

#### In Biofilm Lifestyle With Leaves or Wood as Carbon Sources

*Alnus* leaves and commercial wood-sticks (hazel wood) were macerated overnight in sterile water before being cut in 1 cm^2^ squares which were used both as supports for fungal biofilm development and as carbon sources for *P. cucumerina* AR1. Squares were then sterilized by autoclaving and added in a 250 mL flask containing a ten-fold diluted malt medium (pH 6.5), 0.5 g/L of chloramphenicol without (control; 48 squares) or with 0.25–0.35 mg of *P. cucumerina* AR1 mycelium (96 squares). The flasks were incubated at 28°C under agitation (80 rpm) for 10 days until mature biofilm formation. Then, 16 non-inoculated squares of each substratum were placed into a 250 mL flask containing 100 mL of a more environmentally realistic nicosulfuron concentration of 30 μM in Volvic^®^water (abiotic control). The biofilm-covered squares were incubated in the same way without (growth control) or with 30 μM nicosulfuron.

#### Monitoring of Nicosulfuron Dissipation by *P. cucumerina* AR1

Each of the treatments described above for planktonic and biofilm lifestyles was run in triplicate. The time at which nicosulfuron was added to the planktonic or biofilm-covered substrata cultures was considered as Day 0 (D0). The culture media were sampled at days 0, 3, 6, 10, 14, 21, 28, and 35 to determine herbicide dissipation by HPLC. The production of metabolites was monitored by LC and LC-(+)ESI-MS. At the end of the experiment (day 35), the fungal pellet (planktonic conditions) or one leaf/wood square (biofilm conditions) was extracted in 0.5 mL (leaf) and 2.5 mL (fungal pellet and wood) absolute ethanol to look at sorption onto biomass and/or the substrata. The suspension was stirred vigorously overnight at room temperature and centrifuged (13,000 *g* for 5 min). The extraction was performed twice in order to ensure a complete desorption. The combined organic extracts were concentrated and analyzed by HPLC.

### Identification and Quantification of Nicosulfuron and Its Metabolites

#### Monitoring and Quantification by HPLC

The quantification of nicosulfuron and its main metabolites (ASDM and ADMP), in the culture media (dissipation) and extracted from the fungal biomass, was performed by HPLC on an Agilent Series 1100 chromatograph (Courtaboeuf, France), equipped with a DAD set at λ = 220 and 254 nm, and a reverse phase column (Zorbax Eclipse XDB-C18, 3.5 μm, 75 mm × 4.6 mm) at 22°C. The mobile phase was composed of acetonitrile (Solvent A) and acidified water (H_3_PO_4_, 0.01% v/v; pH 2.9) (Solvent B) at a flow rate of 1 mL min^-1^, linear gradient 0–1 min: 2% A; 1 –10 min: 2–70% A; 10–13 min: 70–100% A; 13 – 13.5 min: 100–2% A; 13.5 – 15 min: 0% A. Injection volume: 5 μL. Each sample was injected twice. Solutions of the commercially available standards (ASDM and ADMP) were prepared in water, by dilution of a mother solution at 1 mM. Each standard solution (covering the expected concentration range) was injected three times. The metabolites N3 and N4 can be quantified only by ^1^H NMR as the standards are not commercially available (Carles et al., 2017a). A “correlation” can be established between the concentrations found by ^1^H NMR and the HPLC area observed. Nevertheless, this correlation is not very accurate. Therefore the precise concentrations for N3 and N4 were not given as they remained very low, in particular for N4.

#### Identification by LC-MS

LC/ESI-MS analyses were performed on a Thermo Scientific UHPLC Ultimate 3000 RSLC coupled with an Orbitrap Q-Exactive analyzer. The crude supernatants were harvested (5 min at 13,000 *g*) before LC-MS analyses and directly injected in the LC-MS system without any further treatment. The analyses were carried out in positive mode. The UHPLC was equipped with a Kinetex EVO C18; 100 x 2.1 mm; 1.7 μm (Phenomenex) at 30°C with a gradient acetonitrile + 0.1% Formic acid (Solvent A) and water +0.1% Formic acid (Solvent B): 0–7.5 min: 5–99% A (linear); 7.5–8.5 min: 99% A; 8.5–9 min: 99–5% A; 9–11 min: 5% A. Flow: 0.45 mL/min. For the mass spectrometer, gaseous N_2_ was used as nebulizer gas (50 L/h). The spray voltage was 3.2 kV. The mass resolution used was 35,000.

### Laccase Activity Measurements

Laccase (EC 1.10.3.2) activity was measured in triplicate at each sampling date from planktonic (varying from 3 to 8 mg of fungal dry mycelium) and biofilm (one square of leaf/wood) samples using the 2,2′-Azino-bis(3-ethylbenzthiazoline-6-sulfonic acid) (ABTS) substrate (Sigma-Aldrich, St. Louis, MO, United States). Enzyme activity assays were conducted according to the protocol of Johannes and Majcherczyk (2000) with some modifications. Substrate saturating conditions were fixed at 3 mM ABTS and incubations were performed during 1 h at 20°C, under agitation (80 rpm) in the dark. ABTS transformation was measured spectrophotometrically (420 nm) using an Ultrospec 2000 device (Pharmacia Biotech, Trowbridge, United Kingdom). The enzymatic activity was expressed as 1 U = 1 μmol ABTS oxidized/g mycelium dry mass/h (ε_420_ = 36 M^-1^ cm^-1^, Johannes and Majcherczyk, 2000). Oven dry mass (DM) was determined systematically for each sample and used to correct laccase activity (activity/h/g DM).

### Biomass Measurements

In the planktonic conditions, fungal biomass production was determined as the dry mass difference between D0 and D35. Biomass corresponding to laccase activity assays was also determined and added to fungal biomass calculations. The biofilm condition did not allow us to calculate a proper fungal biomass production because of the influence of the substrata which was degraded in parallel along the experiment.

### Scanning Electron Microscopy (SEM)

Leaf or wood supports, exposed to nicosulfuron and *P. cucumerina* AR1 strain (biofilm) or not (control), were sampled at D0, D10, D21 and D35 and fixed overnight at 4°C in 0.2 mol/L sodium cacodylate buffer pH 7.4 that contained 1.6 % glutaraldehyde. Biofilms were then washed and post-fixed 1 h with 1% osmium tetroxide in 0.2 mol/L sodium cacodylate buffer (pH 7.4). They were washed 20 min. in distilled water and the dehydration by graded ethanol was performed from 25° to 100° (10 min each) to finish in hexamethyldisilazane (HMDS) for 10 min. After drying, the samples were mounted on stubs using adhesive carbon tabs and sputter-coated with gold-palladium (JFC-1300, JEOL, Japan).

Morphology analysis was carried out using a scanning electron microscope JSM-6060LV (Jeol, Japan) at 5 kV in high-vacuum mode.

### Statistical Analyses

Nicosulfuron dissipation and metabolite production were fitted to an exponential decay model (f = *a* × exp(-*b* × x)) with (*a*) initial nicosulfuron concentration and (*b*) dissipation rate as parameters estimated) and a sigmoidal model (f = *a*/(1+exp(-(x-*x*_0_)/*b*))) with *a* (maximal ADMP or ASDM concentration), *b* (production rate) and *x*_0_ (time when the maximal production rate was achieved) as estimated parameters), respectively, using Sigma Plot 10.0 for Windows (Systat Software, Inc.). Differences on the nicosulfuron dissipation rate and metabolite production rate between treatments were assessed using a one-way ANOVA test followed by Tukey HSD test. ANOVA tests for the planktonic lifestyle experiments used glucose as the fixed factor (10, 5, or 1 g/L), whereas in biofilm lifestyle experiment, it was the substratum (leaf, wood). Before ANOVA testing, data were assessed for normality and homoscedasticity. Log transformations were applied when data did not follow ANOVA assumptions.

## Results

### Characterization of *Plectosphaerella cucumerina* AR1

The fungal spores isolated from submerged *Alnus* leaf communities were fusiform, ends rounded, measuring 8–13 μm in length and 2.5–4 μm in width (Figure 1A). Based on ITS1-5.8S-ITS2-28S region sequencing and on macro- and microscopic characters, the isolated fungus was identified as *Plectosphaerella cucumerina* species and named *Plectosphaerella cucumerina* AR1.

When cultivated in planktonic conditions, the isolated *P. cucumerina* AR1 strain formed threadlike hyphae that grow into a mycelium forming a cottony mass (Figure 1B). Hyphae are septated and produce bud leading to branched mycelium (Figure 1C). Solitary phialides can be produced forming a flask-shaped projection on the apex of the septated hyphae (Figure 1D).

The *Plectosphaerella cucumerina* AR1 colony showed different aspects depending on the solid culture medium used, varying from a white, fluffy and aerial mycelium in Sabouraud chloramphenicol agar medium (Figure 1E) to a beige, smooth in appearance with some white mycelia diffusing from a central dome in PDA medium (Figure 1F). In both cases, the diameter of the colonies reached around 4.5 cm after 14 days at 23°C.

### Biodegradation of Nicosulfuron by Planktonic *P. cucumerina* AR1

*P. cucumerina* AR1 cultivated in mineral medium (MSM) was unable to dissipate 100 μM nicosulfuron (data not shown).

**FIGURE 2:**
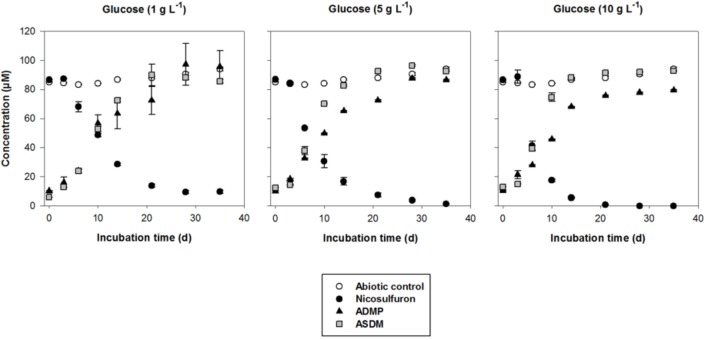
Biodegradation of 100 μM nicosulfuron and production of the major metabolites (ADMP and ASDM) by *P. cucumerina* AR1 strain in planktonic lifestyle in the presence of 1 g/L, 5 g/L, and 10 g/L of glucose. The abiotic controls were non-inoculated with the strain. Values are expressed as the mean ± standard error (SE), *n* = 3.

Nevertheless, when glucose was added as a carbon source, a dissipation of nicosulfuron was observed that follows an exponential decay model (*R*^2^ > 0.91 and *P* < 0.001 for all the conditions tested). This was not the case in abiotic controls. We thus studied the glucose concentration effect, used as a classic co-metabolic substrate, on nicosulfuron biodegradation (Figure 2). The dissipation rates increased with the glucose concentrations (ANOVA, *P* < 0.0001) (Tukey’s test, *P* < 0.05) (Table 1). Nicosulfuron (100 μM) has completely disappeared after 21 days of incubation for a concentration of 10 g/L of glucose, whereas around 1.5% and 13% of nicosulfuron were still remaining after 35 days of culture, for concentrations of 5 and 1 g/L, respectively. Besides, only 2, 1.6, and 1.3% of nicosulfuron were recovered after extraction of the fungal biomass with ethanol after 35 days, when cultures were carried out with 10 g/L, 5 g/L and 1 g/L of glucose, respectively (data not shown). Therefore, biosorption was not a significant process in herbicide dissipation. The degradation of nicosulfuron and growth of the fungal strain did not modify the pH of the culture media which were all measured at 6.50 ± 0.22 (*n* = 18) at the end of the experiment.

**Table 1:** Kinetic parameters of nicosulfuron dissipation and metabolite formation in the presence of various glucose concentrations or natural substrata.

		Dissipation ^(1)^ and production ^(2)^ rates (/h)	SE	*R*^2^	*P*-value
**Glucose**	**Nicosulfuron**				
	Glucose 1 mg/L	0.0739^(1)a^	0.0013	0.9546	<0.0001
	Glucose 5 mg/L	0.1048^(1)b^	0.0061	0.9592	<0.0001
	Glucose 10 mg/L	0.1381^(1)c^	0.0042	0.9139	<0.001
	**Metabolites**				
	ADMP (1 mg/L)	5.3795^(2)a^	0.5782	0.9545	<0.001
	ADMP (5 mg/L)	4.8287^(2)a^	0.0518	0.9882	<0.0001
	ADMP (10 mg/L)	3.9844^(2)a^	0.0920	0.9944	<0.0001
					
	ASDM (1 mg/L)	3.1444^(2)a^	0.0934	0.9895	<0.0001
	ASDM (5 mg/L)	2.9618^(2)ab^	0.0519	0.9923	<0.0001
	ASDM (10 mg/L)	2.5519 ^(2)bc^	0.1298	0.9918	<0.0001
**Natural substrata**	**Nicosulfuron**				
	Leaves	0.1137^(1)a^	0.0021	0.9512	<0.0001
	Wood	0.1139 ^(1)a^	0.0087	0.8815	<0.001


*Parameters are expressed as the mean standard error (SE), *n* = 3. Significant differences between conditions are indicated by letters (Tukey test, *P* < 0.05).*

During the HPLC monitoring of nicosulfuron (*t*_R_ = 7.6 min) biodegradation, two new peaks appeared at shorter retention times (*t*_R_ = 4.3 and 5.2 min) with increasing intensities with time. They were absent from the controls. Analyses of the same samples by LC-(+)ESI-MS gave a molecular ion at m/z 230.0587 [M+H]^+^ (C_8_H_12_N_3_O_3_S^+^) and main fragment ions at m/z 252.0405 [M+Na]^+^ and 213.0323 (C_8_H_9_N_2_O_3_S^+^) for the metabolite with the shortest retention time and a molecular ion at m/z 156.0766 [M+H]^+^ (C_6_H_10_N_3_O_2_^+^) for the second metabolite. According to the literature (Zhao et al., 2015a) and to our previous research work (Carles et al., 2017a), they were assigned to ASDM (2-(aminosulfonyl)-*N,N*-dimethyl-3-pyridinecarboxamide) and ADMP (2-amino-4,6-dimethoxypyrimidine), respectively. The structures of both metabolites were confirmed by comparison with the LC-(+)ESI-MS data of the commercially available standard compounds under the same conditions. These two metabolites are formed by the cleavage of the C-N bond in the sulfonylurea bridge (Supplementary Figure S1). The ASDM production was faster at 10 g/L of glucose compared to 1 g/L (Tukey’s test, *P* < 0.05; Table 1 and Figure 2), accordingly to the results observed with nicosulfuron. Conversely, the production kinetics of ADMP showed no significant difference whatever the glucose concentration tested (Table 1). Nevertheless, both metabolites were present in similar molar concentrations (∼80–90 μM) after 35 days of incubation (Figure 2). Another metabolite, presenting a molecular ion at m/z 347.1456 [M+H]^+^ (C_15_H_19_N_6_O_4_^+^), a retention time at 2.8 min and a main fragment ion at m/z 304.1396 (C_14_H_18_N_5_O_3_^+^), was also detected by LC-(+)ESI-MS in a low amount. It was identified as 2-(1-(4,6-dimethoxy-pyrimidin-2-yl)-ureido)-*N,N*-dimethyl-nicotinamide (N3) (Supplementary Figure S1) (Carles et al., 2017a). Therefore our results indicate that nicosulfuron was mainly co-metabolically degraded by *P. cucumerina* AR1.

**FIGURE 3:**
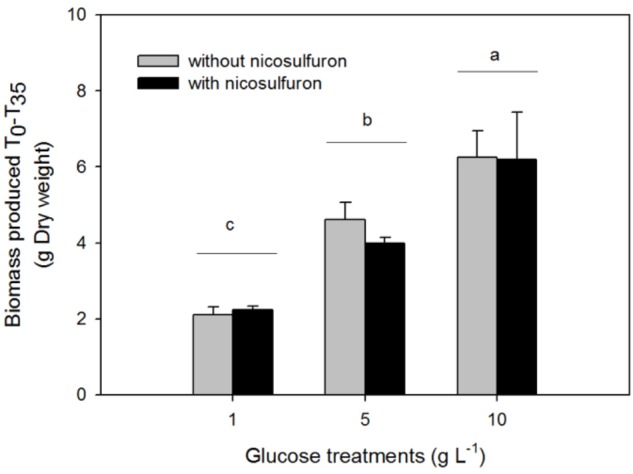
Biomass production of *P. cucumerina* AR1 in the presence of various glucose concentrations and with or without 100 μM nicosulfuron after 35-day incubation. Values are expressed as the mean ± standard error (SE), *n* = 3. Differences between experimental conditions are marked by letters a > b > c (Tukey’s test, *P* < 0.05).

**FIGURE 4:**
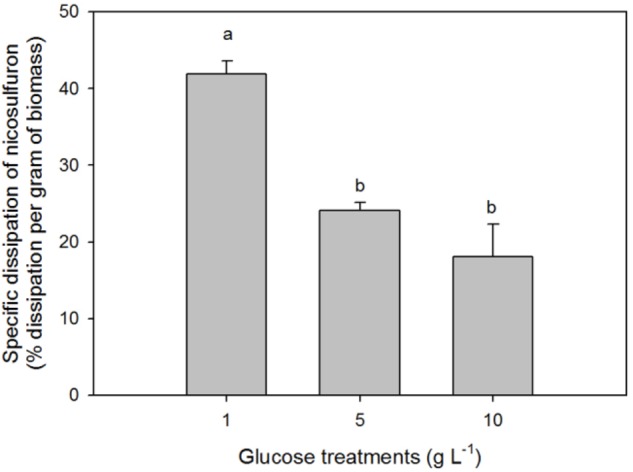
Specific biodegradation percentage of 100 μM nicosulfuron by *P. cucumerina* AR1 in the presence of various glucose concentrations. Values are expressed as the mean ± standard error (SE), *n* = 3. Differences between treatments are marked by letters a > b (Tukey’s test, *P* < 0.05).

The greater the glucose concentration supplied was, the faster the nicosulfuron dissipation (Figure 2 and Table 1). This result can be explained by an increase of mycelium biomass with the increase of glucose concentrations, irrespective of presence of nicosulfuron (ANOVA, *P* < 0.0001, Figure 3). However, specific nicosulfuron dissipation calculations (corrected by mycelium biomass) showed an inverse correlation between specific nicosulfuron dissipation and glucose concentration, varying from 19% at 1 g/L glucose to 8% at 10 g/L (Figure 4).

**FIGURE 5:**
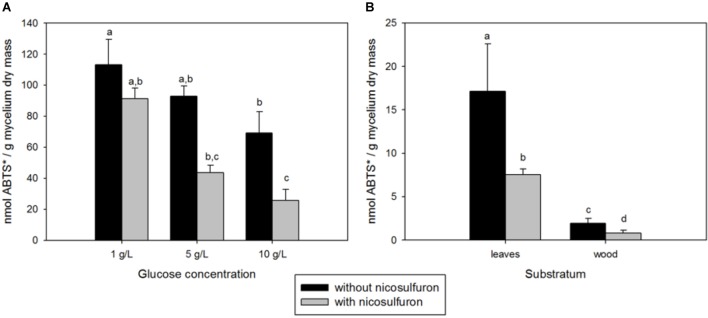
**(A)** Integrated 35-day laccase activity in fungal mycelia grown in the presence or absence of nicosulfuron at different glucose concentrations (1, 5, and 10 g/L). **(B)** Integrated 35-day laccase activity in leaf and wood biofilms supplemented or not with nicosulfuron. Values are expressed as the mean ± standard error (SE), *n* = 3. Differences between experimental conditions are marked by letters a > b > c > d (Tukey’s test, *P* < 0.05).

Although nicosulfuron has no impact on *P. cucumerina* AR1 growth, laccase activity was significantly impaired by both the presence of nicosulfuron and the increasing concentration of glucose (ANOVA, *P* < 0.0001 for both factors; Figure 5A). Only 30% of the laccase activity remained when *P. cucumerina* AR1 was jointly exposed to nicosulfuron and 10 g/L glucose compared to 1 g/L (Tukey’s test *P* < 0.05).

### Biodegradation of Nicosulfuron by Benthic *P. cucumerina* AR1

#### Characterization of the Biofilm Development

**FIGURE 6:**
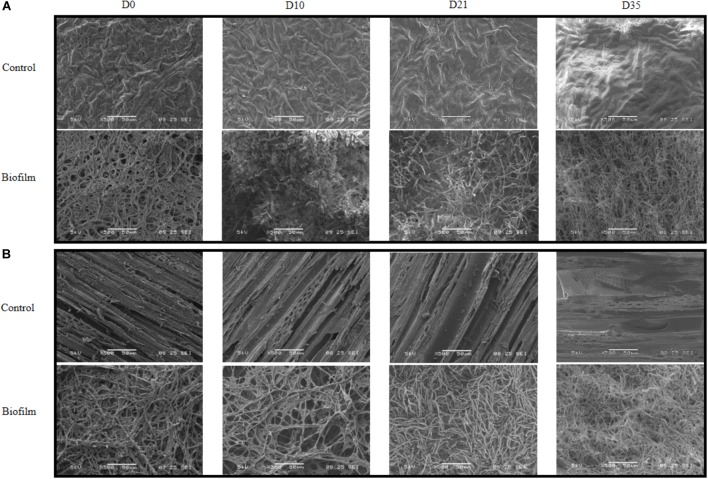
Scanning electron micrographs of leaf **(A)** or wood **(B)** surface exposed to 30 μM nicosulfuron and colonized (biofilm) or not (control) by *P. cucumerina* AR1 strain from day 0 (D0; exposure to nicosulfuron) to the end of the experiment (D35). No contamination of the support was observed for control leaf **(A)** or wood **(B)** supports throughout the experiment (D0–D35). The support surface is completely covered by *P. cucumerina* AR1 biofilm before being exposed to nicosulfuron (D0, **(A,B)**). A fungal development was observed at D10 for leaf **(A)** and D21 for wood **(B)** supports with appearance of a more compact and longer mycelium. Scale bars represent 500 μm.

The *P. cucumerina* AR1 capacity to degrade nicosulfuron in benthic conditions was tested on two natural substrata (alder leaf and hazel wood). The biofilm evolution was monitored by SEM analyses. The SEM micrographs showed no microbial development on leaf and wood supports sterilized by autoclave and incubated in 1/10 diluted malt 1% medium, pH 6.5 (Figures 6A,B control; D0). In contrast, supports inoculated with *P. cucumerina* AR1 presented a well-established biofilm before nicosulfuron addition (Figures 6A,B biofilm; D0).

These biofilms were then exposed to nicosulfuron in Volvic^®^water. The architecture of biofilms evolved slightly differently between leaf and wood substrata. Specifically, a compact and thick biofilm, with rising mycelium, was formed more rapidly on leaves (10 days; Figure 6A biofilm; D10) than on wood (21 days; Figure 6B biofilm; D21). At the end of the experiment, both biofilm structures were comparable (Figures 6A,B biofilm; D35), without apparent differences between control biofilms and those exposed to nicosulfuron.

#### Nicosulfuron Biodegradation

**FIGURE 7:**
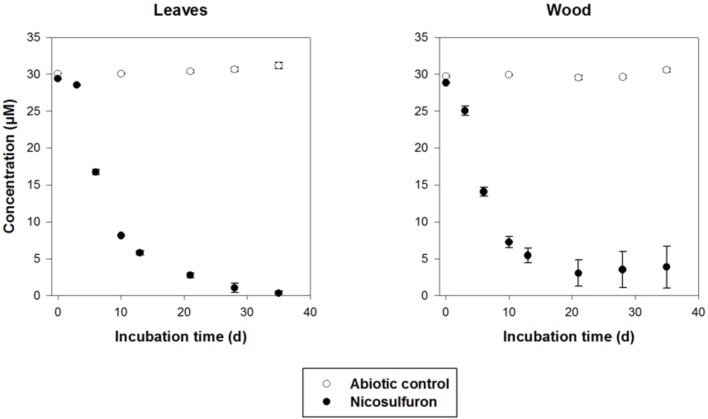
Biodegradation of 30 μM nicosulfuron by *P. cucumerina* AR1 biofilms on leaf and wood. The abiotic controls correspond to the substrata non-inoculated by the strain. Values are expressed as the mean ± standard error (SE), *n* = 3.

The dissipation of nicosulfuron (30 μM) was observed both for leaf and wood grown biofilms (Figure 7). The adsorption of nicosulfuron on subtrata was around 0.15 ± 0.076 % and 2.82 ± 0.69 % (*n* = 3) for leaf and wood, respectively (data not shown). Surprisingly, wood grown biofilms were able to degrade nicosulfuron as soon as they were exposed to the herbicide, as opposed to leaf grown biofilms which showed a 3 day delay in nicosulfuron degradation (Figure 7). Nevertheless, the nicosulfuron degradation kinetics was the same overall, exhibiting comparable rates, whatever the substratum tested (Table 1). At day 21, the nicosulfuron degradation by the wood grown biofilms was maximal, reaching around 97% dissipation. Then, the nicosulfuron concentration remained unchanged until the end of the experiment. On the contrary, for the leaf grown biofilms, the degradation of nicosulfuron reached also 97% after 21 days and continued up to 100% at the end of the experiment (Figure 7). During this biodegradation process, the pH of the medium was not significantly modified since the values were of 6.40 ± 0.36 at the end of the experiment under all the conditions.

Nicosulfuron was degraded by biofilms with the same pathway as that observed for planktonic lifestyle. The two major metabolites, ADMP and ASDM as well as the minor one, N3, were formed under these conditions (data not shown). A fourth metabolite, with a retention time of 2.3 min and a molecular ion at m/z 304.1396 [M+H]^+^ (C_14_H_18_N_5_O_3_^+^), was also detected by LC-(+)ESI-MS after 6 days of incubation but in a very low amount. This ion was already observed in the mass spectrum of N3 as the main fragment ion, suggesting that N4 came directly from N3. It was assigned as 2-(4,6-dimethoxy-pyrimidin-2-yl)-*N,N*-dimethyl-nicotinamide (N4) by comparison with the literature data (Carles et al., 2017a) (Supplementary Figure S1).

During the 35-day experiment, the integrated laccase activity was about 8 times higher for leaf grown biofilms compared to wood grown biofilms (Figure 5B). This activity was decreased (about 55–60%) when biofilms were exposed to nicosulfuron.

## Discussion

*Plectosphaerella cucumerina* AR1 is a filamentous Ascomycete fungus, mostly encountered in the terrestrial environment as a pathogen of various plant species and vegetables [e.g., lettuce (Usami and Katagiri, 2017), cabbage (Li et al., 2017), broomrape (Xu et al., 2016), sunflower (Zhang et al., 2015), bottle gourd (Yan et al., 2016), tomato, melon (Carlucci et al., 2012), potato (Gao et al., 2016)] and to a lesser extent in marine ecosystems where it has been described as host of ascidian invertebrates (López-Legentil et al., 2015), sponges (Wang et al., 2008) and shells (Velmurugan et al., 2011). To our knowledge, this is the first time that *P. cucumerina* (Figure 1) has been isolated from submerged plant litter in a freshwater ecosystem.

Furthermore, *P. cucumerina* AR1 was described as a biological control agent against potato cyst nematodes (Atkins et al., 2003; Jacobs et al., 2003; Dandurand and Knudsen, 2016; Kooliyottil et al., 2017). It was used as a bioherbicide in agricultural crops and pastures (Bailey K. et al., 2017; Bailey K.L. et al., 2017) and would also be involved in the remediation of metal polluted environments (Santelli et al., 2010, 2011).

Our results showed that *P. cucumerina* AR1 can also be used to reduce nicosulfuron contamination since we demonstrated that (i) it was tolerant to nicosulfuron in contrast to what was described for other fungal species (Karpouzas et al., 2014) and (ii) it was able to degrade the nicosulfuron herbicide both in planktonic (Figure 2) and in biofilm conditions with various simple and complex carbon sources (glucose, leaf or wood; Figure 7). Regarding the literature, this is the first time that a leaf-associated fungal strain able to degrade nicosulfuron has been isolated in freshwater, the other ones being isolated from agricultural soil or sludge (Yang et al., 2008; Zhang et al., 2011, 2012; Lu et al., 2012; Song et al., 2013; Zhao et al., 2015a,b, 2018; Wang et al., 2016; Carles et al., 2017a; Feng et al., 2017; Zhou et al., 2017).

As already shown with almost all the isolated nicosulfuron-degrading strains, except the *Oceanisphaera psychrotolerans* LAM-WHM-ZC and *Pseudomonas nitroreducens* NSA02 strains which are able to degrade nicosulfuron in mineral medium, using nicosulfuron as the carbon source (Zhou et al., 2017; Zhao et al., 2018), the nicosulfuron degradation by *P. cucumerina* AR1 was achieved by a co-metabolism process (Figure 2). Our results showed that the herbicide dissipation was mainly due to biodegradation since the pH values of the medium of all the cultures remained around neutrality (ranging from 6.1 to 6.7) at the end of the experiment, contrarily to what was observed with *Penicillium oxalicum* YC-WM1 fungal strain. In this last case, nicosulfuron was degraded by hydrolysis resulting from the acidification of the medium (Feng et al., 2017).

In most cases, the biodegradation rate of a pollutant is improved by the addition of increasing concentrations of the extra carbon source (e. g., Wang et al., 2013; Kirui et al., 2016; Sun et al., 2017) as we observed in our study (Figure 2). However, the specific nicosulfuron biodegradation by the planktonic *P. cucumerina* AR1 was shown to be greater when the concentration of glucose decreased (Figure 4). This phenomenon has already been described for other pollutants (Ye et al., 2011; Shi et al., 2013; Wu et al., 2016). Indeed, the degradation efficiency increased with increasing concentration of glucose until an optimal concentration. Then, the addition of the carbon source to excess inhibited the degradation. This suggests that the optimal glucose concentration for the maximal degradation efficiency of nicosulfuron by planktonic *P. cucumerina* AR1 would be around 1 g/L under the conditions tested (Figure 4).

*Plectosphaerella cucumerina* AR1 used the same nicosulfuron degradation pathway irrespective of its lifestyle, planktonic or in biofilms (Figure 2 and Supplementary Figure S1). It produced two major metabolites, ADMP and ASDM, which were obtained by a pathway common to all the strains described until now, consisting in the biotic hydrolytic cleavage of the sulfonylurea bridge (e.g., Zhang et al., 2012; Song et al., 2013; Zhao et al., 2015a, 2018; Wang et al., 2016; Carles et al., 2017a). The minor N3 metabolite was derived from the cleavage of the C-S bond of the sulfonylurea bridge and contraction by elimination of the sulfur dioxide group, as previously observed with some others nicosulfuron-degrading strains (Song et al., 2013; Zhao et al., 2015a; Carles et al., 2017a; Zhou et al., 2017). Similarly to what was observed during the nicosulfuron degradation by the bacterial strain *Pseudomonas fluorescens* SG-1 (Carles et al., 2017a), the hydrolysis of the N3 urea function lead to the production of the N4 metabolite in small amounts when *P. cucumerina* AR1 was grown in benthic conditions (Supplementary Figure S1).

*Plectosphaerella cucumerina* AR1 strain developed indifferently on both natural substrata studied (alder leaf and hazel wood) (Figure 6). However, the biofilm grew faster on the leaf than on wood. This could be explained by the greater laccase activity rates recorded for the former (Figure 5B). This led to a faster decay of leaves and thus faster nutrient supply for fungal growth comparing to wood substratum which has a more complex molecular arrangement (Golladay and Sinsabaugh, 1991; Gulis et al., 2004). This could also explain the greater laccase activity rates recorded in the leaf substratum (Figure 5B). However, this higher enzyme activity was not correlated with nicosulfuron degradation capacity of *P. cucumerina* AR1, which was similar between leaf and wood biofilms (Table 1). Furthermore, the presence of nicosulfuron did not impact the growth of the fungus since the biofilm development on both substrata was similar to what was observed in the control conditions without herbicide. Similar results were obtained for the planktonic culture conditions for which fungal biomasses were comparable in all experiments, irrespective of the presence of nicosulfuron (Figure 3).

When exposed to nicosulfuron, *P. cucumerina* AR1 biofilms kept the capacity to biodegrade the molecule whatever the substrata tested (Figure 7), thus probably using the decomposition of the natural substrata as nutrients and carbon sources. The present study also shows that nicosulfuron degradation efficiency was greater for *P. cucumerina* AR1 monospecific biofilms on alder leaves (100% dissipation after 28 days, the present study) than for plurispecific natural biofilms hosting *P. cucumerina* on the same leaf species (29–66% dissipation in 40 days, Carles et al., 2017b). The decreased ability of *P. cucumerina* AR1 to dissipate nicosulfuron could be explained either by a relative low presence of the fungus in the natural leaf-associated microbial communities or by microbial interactions within the biofilm. Overall, this is the first time that a benthic strain was shown to be able to degrade nicosulfuron herbicide, all the degradation experiments conducted until now with isolated bacterial and fungal strains being tested in planktonic conditions (Feng et al., 2017; Yang et al., 2008; Zhang et al., 2011, 2012; Lu et al., 2012; Song et al., 2013; Zhao et al., 2015a,b, 2018; Wang et al., 2016; Carles et al., 2017a; Zhou et al., 2017).

The nicosulfuron degradation obtained for *P. cucumerina* AR1 in biofilm conditions showed statistically similar dissipation rates than those observed in planktonic culture conditions containing 5 g/L of glucose (Table 1). This suggests that the natural substrata we provided for co-metabolism reactions could not allow *P. cucumerina* AR1 to degrade the nicosulfuron herbicide at the optimal conditions, since we have shown that a lesser carbon concentration equivalent to 1 g/L of glucose would be more efficient in planktonic conditions. These results still have to be confirmed in biofilm conditions by testing different leaf and wood substrata varying in their composition, and thus in their capacity of decomposition and releasing nutrients (Bani et al., 2018; Bastias et al., 2018).

In contrast to what was often observed in pollutant exposed microbial communities and/or populations (e.g., da Silva Coelho et al., 2010; Artigas et al., 2017; de Araujo et al., 2017; Singh et al., 2017), the laccase activity decreased in the presence of nicosulfuron, showing a reduction of about 60% activity, whatever the lifestyle (Figure 5). The obtained results highlight that laccase activity responses to xenobiotic contamination are probably molecule-specific (Baldrian, 2006).

## Conclusion

We report here the isolation and characterization of a leaf-associated fungus issued from a river ecosystem, identified as a *Plectosphaerella cucumerina* strain. This isolated strain was able to biodegrade the nicosulfuron herbicide by a co-metabolic process. The degradation pathway was shown to be common to almost all the already described nicosulfuron-degrading strains, starting with the hydrolytic cleavage of the sulfonylurea bridge. Nicosulfuron exposure impaired the fungal laccase activity. However, *P. cucumerina* AR1 was able to degrade nicosulfuron in planktonic lifestyle using glucose as the carbon source, with an optimal concentration of 1 g/L. It was also capable of colonizing natural substrata such as alder leaves or hazel wood to form biofilms and to retain its nicosulfuron biodegradation capacity. This suggests that the nutrients and carbon constituting these natural substrata can be used to ensure the co-metabolic reactions and the nicosulfuron dissipation.

Knowing that both leaf and wood surfaces allow the development of extensive biofilms in streams and that fungi are extremely important in their development, the *P. cucumerina* AR1 strain is considered as a potentially useful candidate for the development of methods aiming to reduce contamination by nicosulfuron in aquatic environments.

## Author Contributions

LC and FR set up the experiments, carried out the sampling, and participated to the discussion. CB realized the SEM images. ML carried out the LC-MS analyses. PB-H carried out the HPLC measurements and interpretation. JA isolated the strain and realized the statistical analyses. IB identified the strain. JA and IB designed the experimental work, the cultures of the fungal strain, the laccase and biomass measurements, and wrote the first draft of the article. All the authors wrote sections of the manuscript, participated to the reviewing of the article, and approved the submitted version.

## Conflict of Interest Statement

The authors declare that the research was conducted in the absence of any commercial or financial relationships that could be construed as a potential conflict of interest.
