# Supplementary material for: Nicosulfuron Degradation by an Ascomycete Fungus Isolated From Submerged Alnus Leaf Litter
Source: Front Microbiol. 2018 Dec 19;9:3167. doi: 10.3389/fmicb.2018.03167 (PMC6305708; doi:10.3389/fmicb.2018.03167)
Supplement: Supplementary file 1 [file Presentation_1.PPT]

## Slide 1
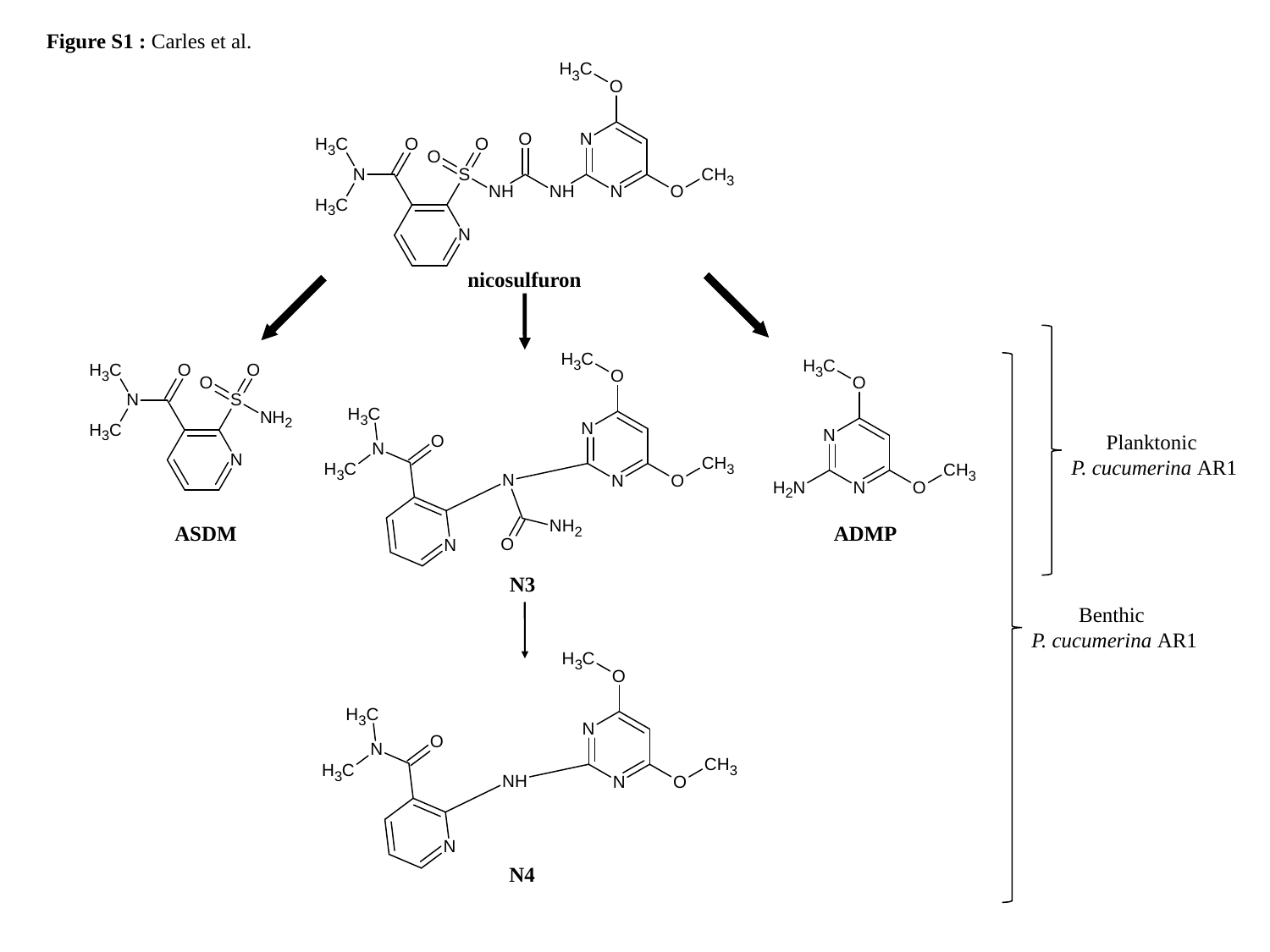

Figure S1 : Carles et al.
nicosulfuron
ASDM
ADMP
N3
N4
Planktonic
P. cucumerina AR1
Benthic
P. cucumerina AR1

## Slide 2
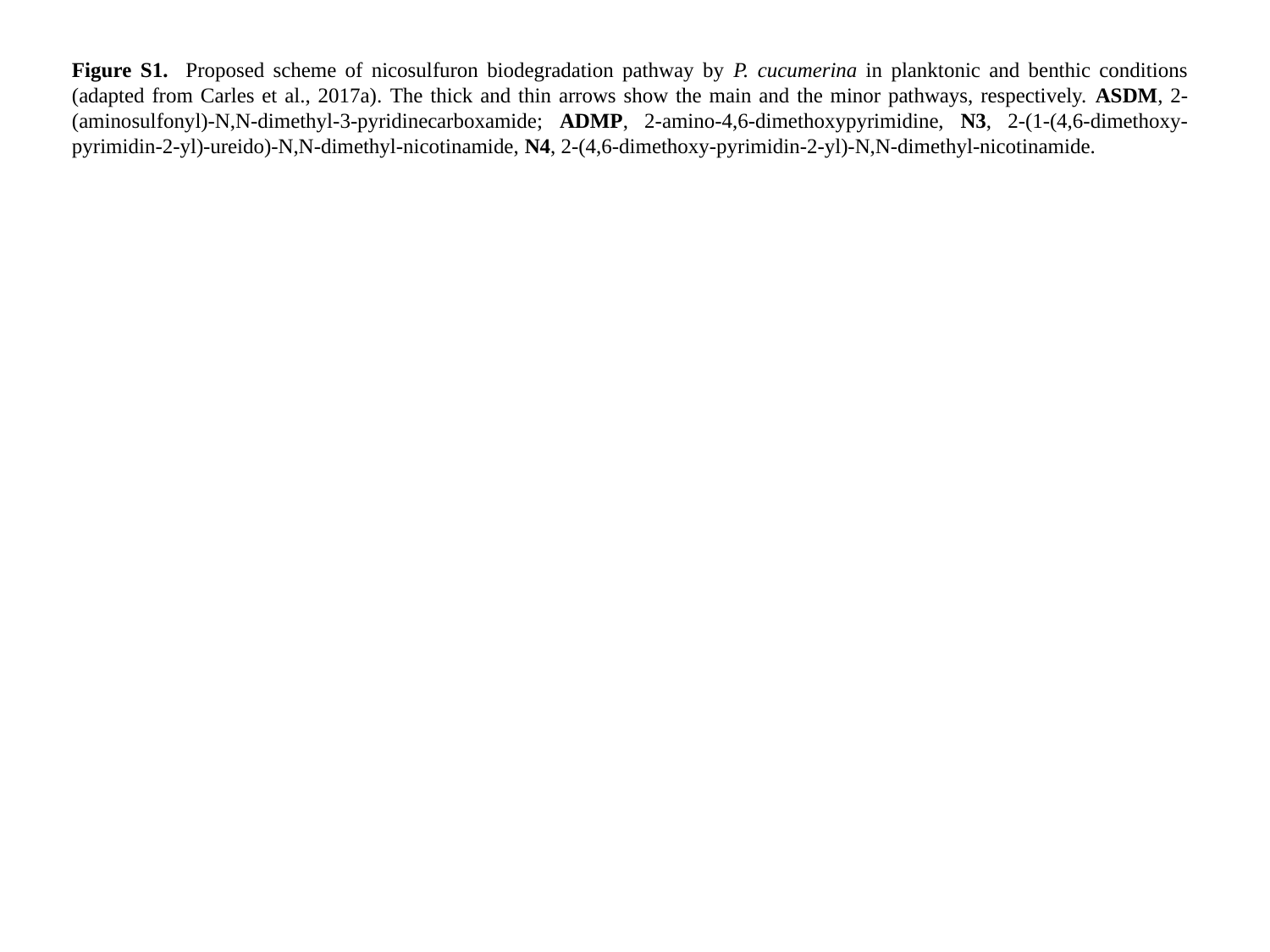

Figure S1. Proposed scheme of nicosulfuron biodegradation pathway by P. cucumerina in planktonic and benthic conditions (adapted from Carles et al., 2017a). The thick and thin arrows show the main and the minor pathways, respectively. ASDM, 2-(aminosulfonyl)-N,N-dimethyl-3-pyridinecarboxamide; ADMP, 2-amino-4,6-dimethoxypyrimidine, N3, 2-(1-(4,6-dimethoxy-pyrimidin-2-yl)-ureido)-N,N-dimethyl-nicotinamide, N4, 2-(4,6-dimethoxy-pyrimidin-2-yl)-N,N-dimethyl-nicotinamide.
